# Major Alterations of Phosphatidylcholine and Lysophosphotidylcholine Lipids in the Substantia Nigra Using an Early Stage Model of Parkinson’s Disease

**DOI:** 10.3390/ijms160818865

**Published:** 2015-08-12

**Authors:** Kyle Farmer, Catherine A. Smith, Shawn Hayley, Jeffrey Smith

**Affiliations:** 1 Carleton University Department of Neuroscience, 1125 Colonel By Drive, Life Sciences Research Building, Ottawa, ON K1S 5B6, Canada; E-Mails: kyle_farmer@carleton.ca (K.F.); csmith@connect.carleton.ca (C.A.S.); 2 Carleton University Department of Chemistry and Institute of Biochemistry, 1125 Colonel By Drive, Steacie Building, Ottawa, ON K1S 5B6, Canada; E-Mail: jeff_smith@carleton.ca

**Keywords:** lipidomic profile, Parkinson’s disease, early stage model, 6-hydroxydopamine, HPLC-ESI-MS/MS

## Abstract

Parkinson’s disease (PD) is a progressive neurodegenerative disease affecting the nigrostriatal pathway, where patients do not manifest motor symptoms until >50% of neurons are lost. Thus, it is of great importance to determine early neuronal changes that may contribute to disease progression. Recent attention has focused on lipids and their role in pro- and anti-apoptotic processes. However, information regarding the lipid alterations in animal models of PD is lacking. In this study, we utilized high performance liquid chromatography electrospray ionization tandem mass spectrometry (HPLC-ESI-MS/MS) and novel HPLC solvent methodology to profile phosphatidylcholines and sphingolipids within the substantia nigra. The ipsilateral substantia nigra pars compacta was collected from rats 21 days after an infusion of 6-hydroxydopamine (6-OHDA), or vehicle into the anterior dorsal striatum. We identified 115 lipid species from their mass/charge ratio using the LMAPS Lipid MS Predict Database. Of these, 19 lipid species (from phosphatidylcholine and lysophosphotidylcholine lipid classes) were significantly altered by 6-OHDA, with most being down-regulated. The two lipid species that were up-regulated were LPC (16:0) and LPC (18:1), which are important for neuroinflammatory signalling. These findings provide a first step in the characterization of lipid changes in early stages of PD-like pathology and could provide novel targets for early interventions in PD.

## 1. Introduction

Parkinson’s disease (PD) is a progressive neurodegenerative disease which affects the dopaminergic nigrostriatal pathway, a brain region critical in the initiation and control of motor behaviour. The degeneration, which occurs over many decades [1] eventually causes severe motor and cognitive deficits [2,3,4]. The primary symptoms of PD are muscle tremors and rigidity within the distal limbs, bradykinesia (slowed movements), and general gait disturbances. Typically PD patients do not begin to manifest primary symptoms until 50%–80% of the nigrostriatum has degenerated [1], resulting in delayed treatments and an overall poorer prognosis. Therefore, identifying early neuronal changes which characterize the early phase of PD (early stage of disease that precedes the manifestation of full blown primary symptoms) is of great importance. Indeed, identifying biomarkers that could help classify the trajectory of illness in PD would greatly aid in the treatment process, as well as provide potential novel targets for the development of future therapeutics.

We have recently utilized a low dose of the specific neurotoxin 6-hydroxydopamine (6-OHDA) to induce a prodromal-like PD state. Specifically, an infusion of 6-OHDA into the dorsal striatum resulted in a modest partial lesion which encompassed 10% and 15% of the striatal volume at two and four weeks after toxin administration, respectively [5]. We also found that our model resulted in no difference in the number of neurons within the substantia nigra (SNc); however, as would be expected in the early stages of PD, many of the neurons had morphology typically seen in unhealthy cells, specifically increased vacuolization, decreased dendritic projections, and an overall decreased cell volume [5]. Additionally, numerous other reports have indicated that pro-death oxidative and mitochondrial stress pathways are likely involved in the early stages of PD, and that pro-inflammatory factors also arise to contribute to primary or secondary pathological processes active in the disease [6,7,8]. At the same time, certain genetic mutations (e.g., LRRK2, Parkin, PINK1) can lead to a familial form of PD or increase the vulnerability to a host of environmental insults, such as pesticides and heavy metals [9,10]. Surprisingly however, scant evidence exists regarding the potential importance of lipid alterations in PD. This is particularly surprising given that many of the toxicants implicated in PD are highly lipid soluble and in fact, accumulate to a substantial degree in brain lipid membranes [11].

Recent attention has focused on lipids and their role in both cell-signalling pathways that govern survival or neurodegeneration. Lysophosphotidylcholines (lysoPC), for example, have been associated with pro-apoptotic processes by activating various deleterious signalling cascades, such as activation of the Bid BH3 protein and caspase-3 [12]. LysoPC species have also been shown to cause decreased expression of the anti-apoptotic TNF receptor-associated factor (TRAF) 2 [13]. Comparatively, sphingolipids (SP) have an interesting dichotomous effect, such that they can act as both a pro-apoptotic and anti-apoptotic second messenger, wherein the difference between the two signalling pathways appears to be dependent on the site of lipid cleavage [14]. Both SP and lysoPC lipid species are derived from phosphatidylcholines (PC), a lipid class which is an important mediator in various structural and signalling roles, having previously been implicated in numerous processes, such as cellular growth and survival [15]. Yet, in many cases it is still unclear as to whether the effects are due to direct signalling by the lipids themselves as second messengers, or due to hyper- or hypoactivity of their associated enzymes [14,15].

In the present investigation, we analyzed the lipid profiles evident in the substantia nigra region of rats subjected to our 6-OHDA early stage model of PD. We then compared the relative quantity and structure of PC, lysoPC and SP using HPLC-ESI-MS/MS. Importantly, we employed a novel HPLC solvent methodology to separate and identify the different lipids according to their individual mass to charge (*m*/*z*) ratio. It is our belief that this broad lipidomics profile can be used as a starting point to investigate the role of lipidomic signaling in the etiology and progression of PD, as well as to further expand research directed at identifying detectable biomarkers of the neurodegenerative condition.

## 2. Results and Discussion

### 2.1. Results

**Figure 1:**
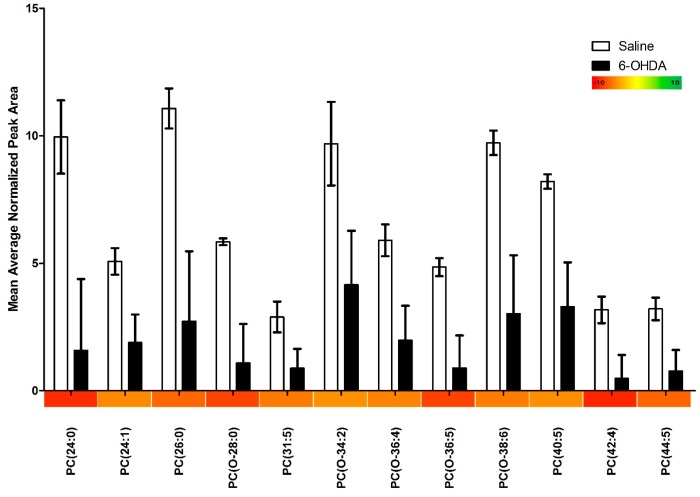
The relative abundance of phosphatidylcholine species in the substantia nigra of animals treated with either 6-OHDA (20 µg) or saline vehicle into the right anterior dorsal striatum, as determined by HPLC-ESI-MS/MS measurements. All measurements were run in triplicate and the error bars represent the standard error. Only species displaying significant differences between treatment and control groups are shown. Significance was determined by independent sample *t*-test. The relative fold change of phosphatidylcholine species is represented along the X-axis in the form of a color heat map.

**Figure 2:**
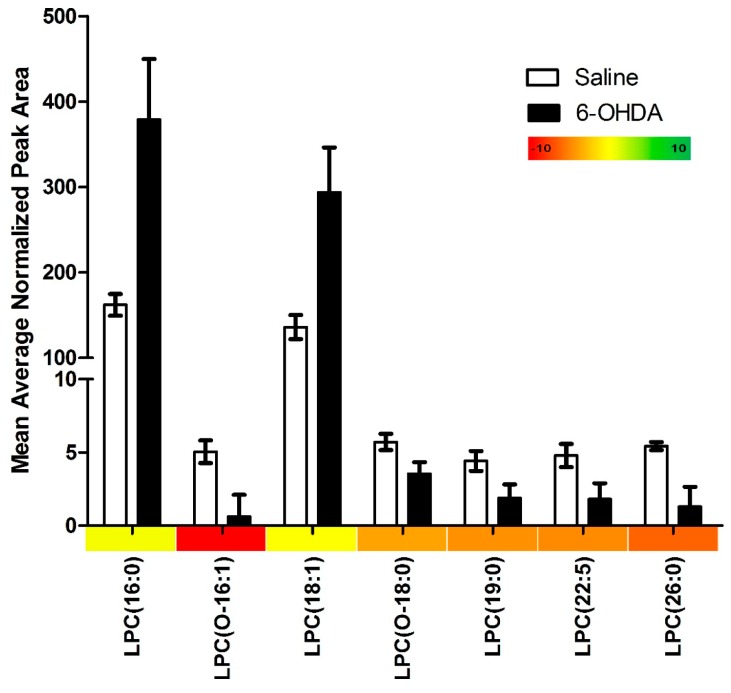
The relative abundance of lysophosphotidylcholine species in the substantia nigra of animals treated with either 6-OHDA (20 µg) or saline vehicle into the right anterior dorsal striatum, as determined by HPLC-ESI-MS/MS measurements. All measurements were run in triplicate and the error bars represent the standard error. Only species that were significantly differences between treatment and control groups are shown. Significance was determined by independent sample *t*-test. The relative fold change of lysophosphotidylcholine species is represented along the X-axis in the form of a color heat map.

Our lipidomics profile scan identified 115 different lipid species, of these, 19 (16.5%) were significantly changed in the 6-OHDA treated animals. We found that 17 (89.5%) of these significantly altered lipids were down-regulated and only 2 (10.5%) were up-regulated. PC species were primarily affected accounting for 63.2% of all significantly altered lipids (12/19), and were found to be entirely down-regulated (Figure 1). Similarly, we found that lysoPC species were mainly down-regulated (Figure 2); however two notable lysoPC lipids were up-regulated; LPC (16:0) and LPC (18:1). The two upregulated lysoPC lipids were also found to be in the highest relative concentrations, with normalized peak area values in the 6-OHDA treated animals being 379 and 294 for LPC (16:0) and (18:1) respectively. Comparatively, the average normalized peak area for all other lysoPC lipids is 1.84, and 1.90 for PC lipids. Finally, we found that 36.8% of the significantly altered lipids contained ether-linked acyl chains. A summary of our findings can be found in Table 1.

**Table 1:** Summary table of lipidomic findings. The class of lipids was determined using the LIPID MAPS MS Prediction Tool.

Category	*N*
Lipids Identified	115
Lipids with Significant Changes	19
Lipids up-regulated in 6-OHDA Animals	2
Lipids down-regulated in 6-OHDA Animals	17
Phosphatidylcholines with Significant Changes	12
Lysophosphatidylcholines with Significant Changes	7

### 2.2. Discussion

In this study we used a relatively low dose of 6-OHDA to induce a partial nigrostriatal lesion, which we believe may partially model a prodromal-like PD state. We have recently found that this animal model causes modest striatal lesions, coupled with morphological abnormalities of surviving neurons [5]. In the present study, we found that PC lipid species were markedly altered in animals treated with 6-OHDA. By far, PC species were the most affected and were generally down-regulated in all cases. Importantly, PC lipids are major components of many cell structures [15], including being present with exceptionally high levels in vacuole membranes [16]. The possibility exists that the decrease of PC lipids in 6-OHDA treated mice reflects some degree of structural rearrangement. In effect, as neurons develop PD like pathology (e.g., decreased dendritic projections, decreased cell volume and cell death) the cell may simply require significantly lower PC lipid concentrations.

In addition to structural support, PC lipids may also contribute to functional aspects of neuronal processes by influencing various signalling pathways. For instance, previous studies have reported that PC lipids modulate anti-inflammatory signaling [17]. Moreover, it is known that 6-OHDA can induce microglial reactivity [5,18,19] and elevations of pro-inflammatory cytokines [20,21,22]. Thus, the decrease in PC lipids currently observed may influence cell survival in the context of 6-OHAD treatment by influencing inflammatory processes.

Although 6-OHDA can provoke pro-apoptotic effects through the protein kinase Cδ (PKCδ) complex system [23], this same system can also impart anti-apoptotic consequences through diacylglycerol (DAG) molecules, which are primary PC lipid metabolites [24,25]. This raises the possibility that the array of lipids modulated by 6-OHDA are likely engaged in a sort of cellular “tug of war” concerning neuronal survival pathways. Another potential source of anti-apoptotic DAG is via sphingomyelin synthase, an enzyme that combines a ceramide and PC lipid to cause the synthesis of a SP and DAG molecule [15]. Although our findings did not show any significant changes in SP lipids, we did find trends in which up-regulations of sphingosine and sphinganine approached significance.

Similar to our PC results, we found an overall decrease in lysoPC species among 6-OHDA treated animals. However, there were two notable exceptions, such that LPC (16:0) and (18:1) were greatly increased in 6-OHDA treated animals. LysoPC are lipids synthesized from PCs via the enzyme phospholipase A2 (PLA2). 6-OHDA has been reported to induce PLA2 enzymatic activity within the nigrostriatal tract [26,27]. Interestingly, increased PLA2 activity has been repeatedly associated with apoptotic effects [28,29] including 6-OHDA induced apoptosis [30]. However the role of PLA2 is exceedingly complex, as the enzyme has also been implicated in anti-apoptotic pathways, primarily via the arachidonic acid and peroxisome proliferator-activated receptor (PPAR) signalling pathway [31]. Arachidonic acid and PPAR system activation has also been shown to reduce oxidative stress and normalize mitochondrial dysfunction [31,32]; both of which are affected by 6-OHDA [33,34,35,36]. Interestingly, both LPC (16:0) and LPC (18:1) have been shown to play major roles in regulating PLA2 enzyme activity by acting as an uncompetitive inhibitor [37]. Alternatively, the accumulation of these LPC species could be related to impairment of other enzymes in the PC re-acylation or de-acylation processes. However, as the literature is lacking on the role of these other enzymes in PD and PD-like pathology, with further enzymology follow up studies required.

It is also possible that the observed increase in LysoPC species (16:0) and (18:1) is unrelated to altered 6-OHDA induced PLA2 enzymatic activity, and instead could be increased to serve a very specific and targeted role by acting as immune signalling mediators. In this regard, it has been shown that LysoPC (16:0) and (18:1) play a major role in inflammatory signaling [37], including the release of various cytokines (interlukin-1β, interlukin-6, chemokine ligand 2, chemokine ligand 4, and tumor necrosis factor alpha) [38,39]. Importantly, the two LPC species have also have potent chemotactic abilities, being able to induce and recruit macrophages and T-lymphocytes to injured tissue [38,40,41,42]. Given these findings, it is possible that the overall decrease of lysoPC species, as a class of lipids, is related to changes in PLA2 activity, whereas, the dramatic increase in LysoPC (16:0) and (18:1) may arise from their involvement in inflammatory processes induced by 6-OHDA. Finally, it is important to point out that we currently report relative differences between the various species rather than absolute lipid levels. Indeed, we are most interested in lipid profile changes that might be characteristic of early stages PD.

In addition to “typical” PC and lysoPC species, we identified numerous “atypical” ether linked forms of these species; all of which were down-regulated. In agreement with our current findings, it has also been reported that PD patients had significantly decreased levels of ether-linked lipids [43]. Ether linked lipids are known to generally be platelet activating factors, and have been shown to be heavily involved in the neuroinflammatory responses [44]. Importantly, drugs that antagonize ether-linked phospholipids have potential clinical relevance given their reported neuroprotective and anti-tumorigenic effects [45,46].

It is of considerable importance to underscore that we currently assessed lipid levels in the SNc, which normally sends projections “downstream” to the striatum to modulate basal ganglia activity. Thus, the observed lipid changes are presumably upstream from the neurotoxin-injected striatum, indicating that they likely stem from retrograde signalling pathways. Indeed, the view is widely held that PD begins with a loss of function and degeneration of the axon terminals in the striatum, which then progresses back to the cell bodies in the SNc [47,48,49,50]. Further to this point, the intra-striatal 6-OHDA model of PD is also known to induce progressive neuronal degeneration over numerous weeks [51,52] and causes increased neuroinflammatory responses [20,22,53]. Additionally, we used a low dose of 6-OHDA which we have recently reported to cause a modest but significant loss of dopamine (DA) striatal terminals, but does not significantly affect the number of neurons within SNc [5]. Taken together, these findings suggest that the intra-striatal 6-OHDA model of PD can be an important tool for the investigation of the early stages of PD as it mimics the human condition in many key aspects.

Mitochondrial dysfunction is an important contributor to PD pathology, and indeed 6-OHDA acts primarily via disruption of mitochondrial processes, resulting in the generation of reactive oxygen species [33,54]. It is important to note, however, that our assay results in the extraction of general cellular and not specific mitochondrial lipids *per se*. Methods to isolate mitochondrial lipids in a manner that renders them amenable to MS analysis are difficult to perform and introduce a great deal of variability.

In the current study, we can only identify the general structure of the various lipid species. Identifying the full fatty acid composition can be achieved with further MS2 analysis using either lithium or acetate adduction methods. However, these methods pose significant limitations, as they are effective for standards, but can be problematic when using complex tissue samples (as they become difficult to separate chromatographically). Both adduction methods are also substantially less sensitive than those involving the production of protonated phosphocholine fragment ions, such as the precursor *m*/*z* 184 scan in positive ion mode (as outlined in the detailed methods section) was used in the current study.

Besides identifying novel lipids that could be mechanistically involved in PD, the present findings could conceivably have implications for the development of biomarkers. Of course, for a molecule to be a useful biomarker it must be readily detectable in peripheral tissue and/or fluids. In this regard, a recent study detected increases in 9 ceramide class lipids in the plasma of PD patients [55]. Moreover, several PC and lysoPC lipid alterations were found in the plasma of patients with Alzheimer’s disease [56]. Future studies are required to assess whether 6-OHDA induces peripheral lipid changes that parallel the presently observed brain lipid changes. However, current evidence is promising and the use of lipidomics screens as a potential tool for early diagnosis of PD may play an important role in the future.

## 3. Experimental Section

### 3.1. Animals

Six male Sprague Dawley rats (Charles River), weighing between 250–280 g on arrival were used in the current experiments. The animals were individually housed in a standard polypropylene cage (27 × 48 × 20 cm) and maintained a 12-h light/dark cycle. Tap water and food (Harlan Rat Chow, Somerville, NJ, USA) was provided ad libitum, while room temperature and humidity were maintained at 20 °C and 50%, respectively. All aspects of this experiment were approved by the Carleton University Committee for Animal Care (P10-28, 3 January 2013) and adhered to the guidelines outlined by the Canadian Council for the Use and Care of Animals in Research.

### 3.2. Surgery

The animals were anaesthetized using variable flow isoflurane inhalational anesthetic (isoflurane volume of 2%–3% in pure O_2_). Animals were then placed in a Kopf instruments Model 940 stereotaxic frame (Kopf Instruments, Tujunga, CA, USA) with the incisor bar positioned 3.3 mm below the incisor line. An L-shaped PlasticsOne 328OP Osmotic Pump Cannula was implanted in the right striatum at the coordinates 1.0 mm anterior, 3.0 mm lateral and 5.0 mm ventral relative to bregma and skull surface. A single infusion of 20 μg of 6-hydroxydopamine (Sigma, St. Louis, MO, USA; purchased as a hydrochloride salt, *n* = 3) in 0.9% injectable saline (containing 0.02% ascorbic acid), or vehicle solution (*n* = 3), was given at a rate of 1 μL/min using a Hamilton 25 μL syringe with a 22 gauge needle attached to a Harvard Apparatus PicoPlus 11 Pump (Harvard Apparatus, Holliston, MA, USA). The vehicle solution was made fresh daily and the 6-hydroxydopamine hydrochloride salt was mixed immediately before the infusion. After the infusion the cannula remained in place for an additional 5 min before being removed to ensure maximal diffusion of the 6-hydroxydopamine solution. BoneWax^®^ (Stoelting, Wood Dale, IL, USA) was placed over the drill hole in the skull and the incision site was clipped using EZclip^®^ surgical staples (Stoelting, Wood Dale, IL, USA). A single application of 2% lidocaine hydrochloride topical anaesthetic jelly (Xylocaine, AstraZeneca, CDMV, Saint-Hyacinthe, QC, Canada) was applied to the incision site.

### 3.3. Euthanasia

Animals were deeply anesthetized with an injection of sodium pentobarbital and subsequently euthanized via transcardial perfusion using a chilled 0.9% saline solution 21 days after the surgical infusion. The ipsilateral substantia nigra (SNc) was isolated from the extracted brain by dissecting the appropriate coronal section using a Kopf Instruments rat blocker obtaining a 2 mm slice approximately −4.3 to −6.3 mm with respect to bregma using the Paxinos and Watson rat atlas [57]. The coronal slice was then further dissected manually using a clean razor blade so as to isolate only the region of tissue containing the substantia nigra. The extracted tissue was immediately flash frozen using 100% ethanol on dry ice. All samples were stored at −80 °C until ready for lipid extraction.

### 3.4. Lipid Extraction

All samples were individually weighed using an analytical balance, measured in grams and accurate to 5 decimal places, to be used later in the data analysis. The extracted tissue was then homogenized via sonication in a solution containing 3.2 mL 0.1 M sodium acetate, 4 mL acidified methanol (2% acetic acid), and 41.3 µL of 10 µM C13:0 LPC (internal standard). Lipids where extracted via liquid–liquid extraction using 3.8 mL of chloroform, centrifuged at 3000 RPM for 2 min at 4 °C. Chloroform extraction was repeated 3 more times using 2 mL chloroform, each replicate being centrifuged at 2000 RPM for 2 min at 4 °C to ensure extraction of all lipids from the original homogenate solution. The extracted chloroform layers were evaporated using ultrapure nitrogen gas, and lipids were subsequently re-suspended in 300 µL absolute ethanol. Lipid samples were stored at −20 °C, and were analyzed via HPLC-ESI-MS/MS within 48 h of being extracted.

### 3.5. HPLC-ESI-MS/MS

Fifteen centimeter lengths of 200 μm inner diameter fused silica (PolymicroTechnologies, Phoenix, AZ, USA) were dipped in a 3:1 solution of Kasil 1678 potassium silicate and formamide (Promega, Madison, WI, USA) to make a column frit on one end. The columns were allowed to dry for 24 h, were shortened to 10 cm, and subsequently packed with 5 cm of 5 μm ReproSil-Pur C4 stationary phase beads (Dr. Maisch GmbH, Ammerbuch, Germany) in acetone using a nitrogen pressure vessel. A PicoFrit Emitter (New Objective, Woburn, MA, USA) was filled in a similar manner, however only 1 cm of ReproSil-Pur C4 beads were used.

Test samples were prepared immediately before each run (1 μL of concentrated lipid sample, 5 μL of absolute ethanol, and 34 μL of deionized water). Samples were placed into a Dionex UltiMate 3000 autosampler (Thermo Fisher Scientific, Waltham, MA, USA) maintained at 4 °C and loaded onto the chromatography column using a Dionex UltiMate 3000 pump (Thermo Fisher Scientific, Waltham, MA, USA, 20 µL total volume). All mobile phases were prepared using HPLC grade solvents: (A) 30% methanol in 10 mM ammonium acetate; (B) isopropanol with 10 mM ammonium acetate and (C) hexane. Each lipid mass analysis was 60 min in length and began with a 100% A mobile phase. Four min later solvent B was gradually increased until the mobile phase became 100% B at the 45.5 min mark where is remained at 100% B for 1 min. At the 46.6 min mark solvent C gradually increased until the mobile phase became 100% C where it remained until the end of the run.

Chromatographically separated lipids were directly analyzed using an AB Sciex 4000 QTRAP ESI-MS/MS Hybrid Triple Quadrupole/Linear Ion Trap (AB Sciex, Framingham, MA, USA). The QTRAP was run in positive ion mode, with the ESI nanospray voltage set at 3 kV, curtain gas at 20 and declustering potential at 25 V. A precursor ion scan and an enhanced mass spectrum (EMS) were conducted using a mass range of 250–1500 Da. The precursor ion scan was set to analyze precursors of *m*/*z* 184 with a collision energy of 40.0 eV and collision cell exit potential of 10 V.

Immediately after each lipid sample was analyzed, a 140 min hexane wash was run (90% C and 10% B). Towards the end of the wash at the 130 min mark the mobile phase switched to 100% B and before switching to 100% A at 137 min. A 40 min blank was run immediately following the wash cycle to ensure there was no carry over. The mobile phase began with 100% A before gradually switching to 100% B at starting a 4 min and ending at 28 min. The mobile phase remained at 100% B for another minute before returning to 100% A until the end of the run. All samples were run in triplicate.

### 3.6. Data Analysis

Mass spectra were analyzed using Analyst Software version 1.5.1 (AB Sciex, Concord, ON, Canada). The precursor *m*/*z* 184 scan was manually analyzed to generate a list of phosphatidylcholine and sphingolipid *m*/*z*-values. The *m*/*z*-values (±0.2 Da) were then entered into MultiQuant 2.1.1 (AB Sciex, Concord, ON, Canada). All desired peaks were integrated and an output containing a variety of parameters, such as peak area and retention time, was generated. The peak area of each lipid was divided by the peak area of the internal standard C13:0 LPC (*m*/*z* 454), and then again by the total weight of the substantia nigra. The triplicate data was averaged for each animal providing a biological *n* of 3 per treatment group. LIPID MAPS MS Prediction Tool (LIPID MAPS, San Diego, CA, USA) was then used to determine the classification and to predict the structure of all lipid masses identified in the precursor *m*/*z* 184 scan. Statistical significance between treatment groups for each lipid species was determined using independent-samples *t*-tests.

## 4. Conclusions

We report that 21 days after an intrastriatal infusion of a low dose of 6-OHDA there are significant decreases in PC and lysoPC within the SNc. Two lipids, LysoPC (16:0) and (18:1) stood out in that they greatly upregulated by 6-OHDA. Although speculative, these observed lipid changes may contribute to structural reconfiguration, immune system signalling, and pro- and anti-apoptotic pathways. This broad lipidomic profile is novel and might be used to further investigate the role of specific lipid species in the etiology and progression of PD, as well as providing potential biomarkers for PD progression. Of course, to be ultimately useful in this capacity, future work will be required to determine whether peripheral lipid species change in parallel with the presently observed SNc variations. Finally, it is particularly noteworthy that the series of marked lipid changes occurred within the SNc, which is of course, up-stream of the 6-OHDA injected striatum. Thus, our findings clearly indicate that retrograde signals likely emanated from the partially lesioned striatum to modulate lipid composition within the SNc cell soma.
